# Prediction model of random forest for the risk of hyperuricemia in a Chinese basic health checkup test

**DOI:** 10.1042/BSR20203859

**Published:** 2021-04-07

**Authors:** Yuhan Gao, Shichong Jia, Dihua Li, Chao Huang, Zhaowei Meng, Yan Wang, Mei Yu, Tianyi Xu, Ming Liu, Jinhong Sun, Qiyu Jia, Qing Zhang, Ying Gao, Kun Song, Xing Wang, Yaguang Fan

**Affiliations:** 1 Department of Nuclear Medicine, Tianjin Medical University General Hospital, Tianjin, P.R. China; 2 School of Medical Imaging, Tianjin Medical University, Tianjin, P.R. China; 3 School of Medicine, Shanghai Jiao Tong University, Shanghai, P.R. China; 4 Tianjin Key Laboratory of Acute Abdomen Disease Associated Organ Injury and ITCWM Repair, Institute of Acute Abdominal Diseases, Tianjin Nankai Hospital, Tianjin, China; 5 Senior Lecturer in Statistics, Hull York Medical School, University of Hull, Hull, U.K.; 6 Tianjin University of Traditional Chinese Medicine, Jian Kang Chan Ye Yuan, Jinghai District, Tianjin, P.R. China; 7 College of Intelligence and Computing, Tianjin Key Laboratory of Advanced Networking (TANK Lab), Tianjin Key Laboratory of Cognitive Computing and Application, Tianjin, China; 8 Department of Endocrinology and Metabolism, Tianjin Medical University General Hospital, Tianjin, P.R. China; 9 Department of Health Management, Tianjin Medical University General Hospital, Tianjin, P.R. China; 10 Tianjin Key Laboratory of Lung Cancer Metastasis and Tumor Microenvironment, Tianjin Lung Cancer Institute, Tianjin Medical University General Hospital, Tianjin, P.R. China

**Keywords:** Hyperuricemia, prediction model, random forest

## Abstract

Objectives: The present study aimed to develop a random forest (RF) based prediction model for hyperuricemia (HUA) and compare its performance with the conventional logistic regression (LR) model. Methods: This cross-sectional study recruited 91,690 participants (14,032 with HUA, 77,658 without HUA). We constructed a RF-based prediction model in the training sets and evaluated it in the validation sets. Performance of the RF model was compared with the LR model by receiver operating characteristic (ROC) curve analysis. Results: The sensitivity and specificity of the RF models were 0.702 and 0.650 in males, 0.767 and 0.721 in females. The positive predictive value (PPV) and negative predictive value (NPV) were 0.372 and 0.881 in males, 0.159 and 0.978 in females. AUC of the RF models was 0.739 (0.728–0.750) in males and 0.818 (0.799–0.837) in females. AUC of the LR models were 0.730 (0.718–0.741) for males and 0.815 (0.795–0.835) for females. The predictive power of RF was slightly higher than that of LR, but was not statistically significant in females (Delong tests, *P*=0.0015 for males, *P*=0.5415 for females). Conclusion: Compared with LR, the good performance in HUA status prediction and the tolerance of features associations or interactions showed great potential of RF in further application. A prospective cohort is necessary for HUA developing prediction. People with high risk factors should be encouraged to actively control to reduce the probability of developing HUA.

## Introduction

Uric acid (UA) is the final metabolite of purines in human. Hyperuricemia (HUA) may result from under-excretion or over-production of UA in congenital or acquired ways [1]. According to the National Health and Nutrition Examination Survey (NHANES) 2015–2016 in the U.S.A., the prevalence of HUA was 20.1% (20.2% and 20.0% in males and females) and was stable during the last decade (*P*=0.24) [2]. A meta-analysis conducted in China, which pooled study results from 2000 to 2014, showed the prevalence of HUA in China was 13.3% (19.4% and 7.9% in males and females) [3]. A higher prevalence of 16.4% was reported in a separate meta-analysis pooling study from 2000 to 2019 in China (20.4% and 9.8% in males and females) [4]. Although lower than the U.S.A., the prevalence of HUA in China has doubled during the last two decades,making it another common metabolic disease after diabetes mellitus (DM).

Extensive evidence has shown that, in addition to triggering of gout, elevated serum UA is an independent risk factor for chronic kidney disease, hypertension, cardiovascular diseases, dyslipidemia and impaired glucose metabolism, as it also plays an important role in the premature mortality observed in the diseases [5,6]. It has also been demonstrated that patients with asymptomatic HUA may have urate deposition over joints or even bone erosion, suggesting that asymptomatic HUA to gout is a continuous pathological process [7,8]. Therefore, it is of great significance to predict HUA early and conduct secondary prevention for high-risk groups.

Thus far, there are few prediction models for HUA. Cao et al. developed a Cox regression model using routine anthropometric and blood biomarkers in urban Han Chinese adult [9]. Zeng et al. developed an artificial neural network prediction model in Chinese adults based on dietary risk factors [10]. Lee et al. tried several machine learning algorithms to predict HUA status in Korea people over 40 [11]. As a good clinical prediction model, it should have the characteristics of high prediction power, easy-understanding and convenient-operating. Random forest (RF) is an algorithm that integrates multiple decision trees through the idea of ensemble learning and is capable of representing high order interactions. With further research on HUA, predictors of the model can also be supplemented and replaced without worrying about interaction or association between variables. In the present study, based on the RF model, we established a gender-specific prediction model for HUA, and compared its performance with the conventional logistic regression (LR) model, with the aim of developing a predictive model that can be easily generalized and preventing more further adverse health consequences from HUA.

## Methods

### Study population

The study protocol was approved by the Institutional Review Board and Ethics Committee of Tianjin Medical University General Hospital, China (IRB number 2011-6-1). We analyzed a previously constructed database from a basic health check program during September 2011 to April 2014. About 148,210 subjects underwent the health check program. Cases with any vacant data were deleted, and a total of 91,690 (83.56%) subjects were invited into this cross-sectional study.

### Measurements and definitions

Anthropometric measurements and blood tests of the participants were performed during their visits to our institutions. Height and weight were measured in centimeters and kilograms. Body mass index (BMI) was calculated by dividing weight (kg) by the square of height (m^2^). Waist circumference (WC) was measured in centimeters. Systolic and diastolic blood pressure (SBP/DBP) were measured by an automated sphygmomanometer while the subjects were in a seated position after resting for 5 min. Blood tests were measured by an auto-analyzer (Hitachi Model 7600 analyzer, Hitachi, Tokyo, Japan). Alcohol intake and smoking history were from the self-recorded questionnaires.

The diagnostic criteria for HUA was SUA>420 μmol/l in males and >360 μmol/l in females [12]. The diagnostic criteria for DM was fasting plasma glucose ≥7.0 mmol/l [13].

### Statistical analysis

We tested all parameters for the normality by the Kolmogorov–Smirnov test. The subject characteristics were assessed using Mann–Whitney *U* test. Differences were considered significant at *P*<0.05. The results were represented as median (quartile 1-quartile 3), otherwise stated. Odds ratio (OR) with 95% confidence interval (CI) was calculated by the LR. Statistical analysis was performed using SPSS version 26 (IBM Corporation). Comparison of the receiver operating characteristic (ROC) curves was done with the Delong test using MedCalc version 19.

### RF model

In the present study, tree parameters trained in male dataset included ‘criterion’ as ‘entropy’, ‘max_depth' as 10, ‘min_samples_leaf’ as 12 and ‘n_estimators’ as 70; and in female dataset included: ‘criterion’ as ‘gini’, ‘max_depth’ as 10, ‘min_samples_leaf’ as 12 and ‘n_estimators’ as 90. Models were developed using Python 3.7.

### Model evaluation

The discriminatory power of models was analyzed by ROC curves. Sensitivity, specificity, positive predictive value (PPV) and negative predictive value (NPV) were calculated. Cutoff score with the maximum sum of sensitivity and specificity was considered optimum [14].

## Results

### Studying characteristics

A total of 91,690 subjects were included in this cross-sectional study. The cohort was randomly divided into a training set (ratio of 0.8, *n*=73,351) and a validation set (ratio of 0.2, *n* = 18,339). Since the number of non-HUA was much larger than HUA, simple down-sampling was carried out on the non-HUA set based on the sample size of HUA to adjust for data imbalance. As a result, the sample distribution of the training sets was 9077/8986 in males and 2054/2033 in females (HUA/non-HUA). The validation set retained the real-world population distribution. The distribution of the validation sets was 2399/8123(29.53%) in males and 502/7315(6.86%) in females (HUA/non-HUA).

A total of 21 variables including anthropometric measurements, blood tests, alcohol intake and smoking history were firstly examined in the RF model (shown in the Supplementary Material), of which the top 10 with the highest weight were selected for each gender (Table 1). All variables differed significantly between groups (*P*<0.001). About 8/10 variables were consistent across gender models. Among HUA patients, the distribution of age and TC were higher in female (*P*<0.001), and the other variables were significantly higher in male (*P*<0.001).

**Table 1:** Baseline characteristics with the top ten weight value in validation set

Variables	Non-HUA	HUA	*P* value	Weight
**Males**				
TG	1.34 (0.95–1.96)	1.83 (1.28–2.63)	<0.001	0.16
Cr	78.00 (71.00–85.00)	82.00 (75.00–90.00)	<0.001	0.14
ALT	22.00 (17.00–32.00)	28.00 (20.00–43.00)	<0.001	0.09
BMI	25.30 (23.20-27.50)	26.90 (24.90–29.10)	<0.001	0.09
Weight	74.80 (67.60-82.10)	80.20 (73.00–88.40)	<0.001	0.08
Age	46.00 (35.00-57.00)	41.90 (32.00–53.00)	<0.001	0.07
WC	88.00 (82.00-94.00)	92.00 (87.00–98.00)	<0.001	0.06
TC	5.01 (4.42–5.65)	5.27 (4.62–5.92)	<0.001	0.05
FPG	5.00 (4.60–5.50)	5.10 (4.70–5.50)	<0.001	0.04
WBC	5.70 (4.90–6.70)	6.00 (5.20–6.90)	<0.001	0.03
**Females**				
TG	0.95 (0.68–1.39)	1.52 (1.04–2.21)	<0.001	0.16
Cr	59.00 (53.00–64.00)	66.00 (58.00–72.25)	<0.001	0.13
BMI	22.90 (20.80–25.40)	26.20 (23.30–29.20)	<0.001	0.12
WC	76.00 (70.00–83.00)	85.00 (77.75–92.25)	<0.001	0.08
ALT	15.00 (12.00–20.00)	19.00 (15.00–28.00)	<0.001	0.07
Weight	58.90 (53.50–65.30)	65.85 (59.00–73.23)	<0.001	0.06
BU	4.30 (3.50–5.00)	5.00 (4.20–5.90)	<0.001	0.06
Age	43.00 (31.00–55.00)	55.45 (34.98–65.83)	<0.001	0.05
SBP	115.00 (105.00–130.00)	130.00 (115.00–145.00)	<0.001	0.04
TC	4.99 (4.35–5.71)	5.42 (4.68–6.21)	<0.001	0.04

Abbreviations: ALT, alanine aminotransferase; BMI, body mass index; Cr, creatinine; FPG, fasting plasma glucose; G, triglyceride; SBP, systolic blood pressure; TC, total cholesterol; WBC, white blood cell; WC, waist circumference. Kolmogorov–Smirnov test showed *P* values of all data were less than 0.001. Mann–Whitney *U* test was carried out in all variables. Results were represented as median (the first quartile – the third quartile). Feature value was rounded to two decimal places.

### Evaluation of RF model predictive ability

Classification matrix of the validation set was shown in Table 2. The sensitivity and specificity of the RF model were 0.702 and 0.650 in males, 0.767 and 0.721 in females. PPV and NPV were 0.372 and 0.881 in males, 0.159 and 0.978 in females. Area under the curve (AUC) of the RF models was 0.739 (0.728–0.750) in males and 0.818 (0.799–0.837) in females.

**Table 2:** Classification matrix in different gender

	Males			Females		
Case status	Prediction classification			Prediction classification		
	HUA	Non-HUA	Total	HUA	Non-HUA	Total
HUA	1685	714	2399	385	117	502
Non-HUA	2839	5284	8123	2040	5275	7315
Total	4524	5998	10522	2425	5392	7817

### Evaluation of LR model predictive ability

**Figure 1:**
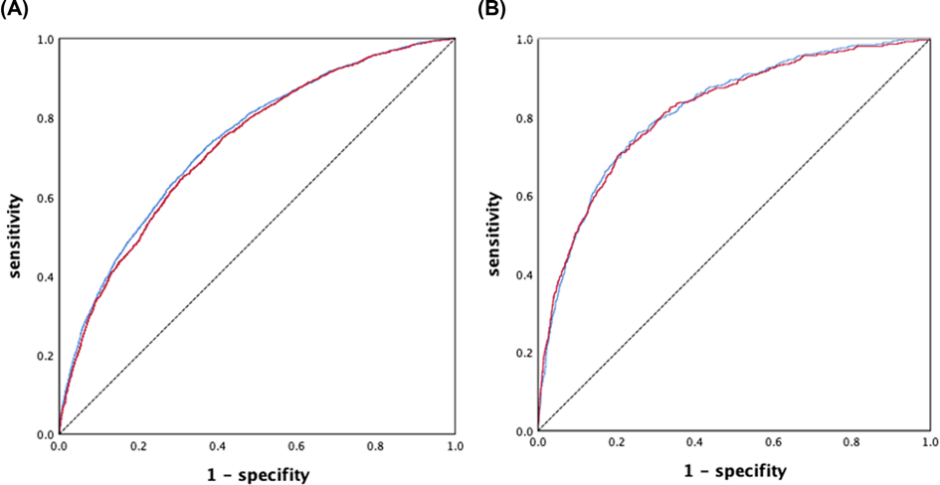
Discriminatory power of the two models The figures showed the discriminatory power of RF (blue) and LR (red) for HUA ((**A**) for males; (**B**) for females). AUC of RF was higher in RF in both genders. Delong tests showed the significance in males.

We further incorporated these parameters into the conventional LR model. First, we conducted univariate LR in both gender groups and all variables except FPG showed statistical significance (Table 3). Next, we conducted multiple LR for both. Since LR model is sensitive to the collinearity of variables, we screened the 10 variables in model 1 with tolerance = 0.2 as the cutoff in each gender. For males, BMI, weight and WC suggested significant collinearity so that we only left one. Multiple LR suggested that variables except WBC were of statistical significance in BMI-retained model (model 2). Interestingly, all variables were shown to be statistically significant when weight was the one to be retained (not shown in the table). For females, collinearity was shown between weight and BMI. The BMI-retained model suggested that WC was not statistically significant, while weight-retained model showed a contradictory result. AUC of the LR models were 0.730 (0.718–0.741) for males and 0.815 (0.795–0.835) for females (Figure 1). The predictive power of RF was slightly higher than that of LR, although it was not statistically significant in females (Delong tests, *P*=0.0015 for males, *P*=0.5415 for females).

**Table 3:** Analysis of risk factors on HUA based on logistic regression model

Sex/Variables	Model 1		Model 2	
	Crude OR (95%CI)	*P*	Adjusted OR (95%CI)^*^	*P*
**Males**				
TG	1.288 (1.247–1.330)	<0.001	1.207 (1.165–1.251)	<0.001
Cr	1.037 (1.033–1.041)	<0.001	1.043 (1.038–1.047)	<0.001
ALT	1.016 (1.014–1.018)	<0.001	1.007 (1.005–1.009)	<0.001
BMI	1.161 (1.145–1.177)	<0.001	1.136 (1.118–1.153)	<0.001
Weight	1.046 (1.042–1.050)	<0.001	–	
Age	0.985 (0.981–0.988)	<0.001	0.981 (0.977–0.984)	<0.001
WC	1.053 (1.047–1.058)	<0.001	–	
TC	1.228 (1.229–1.348)	<0.001	1.163 (1.102–1.228)	<0.001
FPG	0.969 (0.932–1.007)	0.112	0.871 (0.830–0.913)	<0.001
<7.0			1.065 (0.977–1.162)	0.154
≥7.0			0.811 (0.711–0.926)	0.002
WBC	1.147 (1.112–1.183)	<0.001	1.027 (0.992–1.064)	0.135
**Females**				
TG	1.596 (1.476–1.726)	<0.001	1.302 (1.202–1.410)	<0.001
Cr	1.068 (1.059–1.078)	<0.001	1.061 (1.051–1.072)	<0.001
BMI	1.229 (1.201–1.258)	<0.001	1.172 (1.120–1.227)	<0.001
WC	1.076 (1.067–1.086)	<0.001	1.005 (0.986–1.023)	0.612
ALT	1.021 (1.016–1.026)	<0.001	1.010 (1.005–1.015)	<0.001
Weight	1.071 (1.062–1.080)	<0.001	–	
BU	1.595 (1.493–1.705)	<0.001	1.263 (1.164–1.370)	<0.001
Age	1.033 (1.027–1.039)	<0.001	0.987 (0.978–0.995)	0.002
SBP	1.032 (1.027–1.036)	<0.001	1.010 (1.004–1.016)	0.001
TC	1.452 (1.339–1.575)	<0.001	1.062 (0.960–1.175)	0.240

* Adjusted with other 9 selected covariates.

## Discussion

In this study, we obtained a balanced training set by simple down-sampling, then developed a gender-specific RF model to predict the status of HUA and compared its performance with the most commonly used LR model. It showed that the predictive power of RF was higher than LR in both genders, although it was not significant in females.

Frequently the classifiers based on medical data are developed using class-imbalanced data, where the number of subjects with a particular disease is far less than the healthy. Our study contained 91,690 participants, of which 14,032 were HUA and 77,658 were non-HUA. Standard classification methods applied to class-imbalanced data tend to produce classifiers biased toward the majority class [15]. Therefore, increasing attention has been paid to between-class imbalance problem to improve the accuracy of the minority class prediction [16]. However, practice of imbalanced data processing is not widely used in clinical practice [17]. In this study, based on our relatively sufficient sample size, we tried simple down-sampling to ameliorate the data imbalance [17].

Machine learning has been popular in various fields for years. Due to its complexity in understanding and operation, it has not been fully applied in the clinical work. RF, as an ensemble learning algorithm, integrates multiple decision trees, of which each tree depends on the values of a random vector sampled independently and with the same distribution for all trees in the forest [18]. Compared with the conventional statistical methods, it has the following advantages: (1) RF, as a high-throughput algorithm, is able to handle high-dimensional features without dimension reduction; (2) RF gives the weighted value of each feature in the classification so that the contribution of variables are comparable; (3) RF can deal with imbalanced or missing data to some extent; (4) Interaction or association between variables does not impact much on the classification[19].

In this paper, we tried two different methods as RF and LR to predict the status of HUA. The variables in the RF model were significantly different between HUA and non-HUA (Table 1), which strengthened the theoretical basis and practical confidence for further HUA developing prediction model. The model developed by RF showed high value in predicting HUA. AUC of the RF models was 0.739 (0.728–0.750) in males and 0.818 (0.799–0.837) in females. Note that the PPVs in both genders were not high, which may be related to the prevalence of HUA in the population. When sensitivity and specificity in a diagnostic test remained unchanged, higher PPV will be obtained in population with a higher prevalence. However, due to the relatively low prevalence in the real-world data, the true positive sets of the diagnostic test do not increase significantly even if the sensitivity increases, so the method based on adjusting sensitivity has little effect on improving the positive predictive value. By increasing specificity can significantly reduce the nsizeof false positive sets, so as to improve the PPV. However, the mpresent odel is for screening HUA in the population so that the sensitivity should not be abandoned while the specificity is improved. Therefore, we made a trade-off in our study and chose the cutoff point with the maximum sum of sensitivity and specificity.

Comparing with LR, we found that the AUC of RF was higher in both gender models, although it was not significant in females. However, as mentioned above, any interaction or association between variables does not adversely affect the RF classification while it does in LR. Since LR is easily affected by the collinearity of variables, when building the LR model, we took the tolerance = 0.2 as the bound value, and only one variable in the collinearity set was reserved. We found that when retaining different variables, the effect on the remaining variables was different. Indeed, when one confounding factor is adjusted for, any relevant variable will also be changed. In this case, the choice about the retained variables becomes difficult, especially when there are many variables. At this point, it is methodologically better to use RF, for which is not that sensitive to collinearity. To sum up, we believe that RF has great potential in HUA prediction.

As for the risk factors, in addition to indicators discussed in previous studies, we found that the role of FPG is worth mentioning. FPG ranked 10th in males and 15th in females. After adjusting other covariates, there was no significant association between FPG level and HUA in normal FPG group (*P*=0.154), but a negative association was found in DM group in males (OR = 0.811, *P*=0.002), which was in consistent with previous studies [20]. Previous studies have indicated that diabetic patients with glycosuria had a null prevalence of HUA and excreted more UA than those without glycosuria [21]. Chino et al. showed that glycosuria induced by SGLT2 inhibitors at the proximal tubule might inhibit UA reabsorption [21]. Glycosuria resulting from elevated glucose levels in diabetic patients might lead to a competitive inhibition of UA reabsorption. However, the relationship between FPG and serum UA still remains controversial. Studies have reported that serum UA can cause pancreatic β-cell dysfunction, so as HUA becomes an independent risk factor for Type 2 DM [22,23]. In addition, the weight of FPG in female classification is relatively low, and whether there is an interaction between FPG and hormones still needs to be further explored.

The study also has some limitations. First, the dataset is based on a single-center cross-sectional study, and there may be selection bias. Second, underlying disease of participants from the program could not be as detailed as the hospital records, which might introduce confounding factors.

## Conclusions

In conclusion, we developed a RF prediction model of HUA status based on a cross-sectional data, and the model achieved good results. Compared with the LR, RF has its own irreplaceable advantages, and we believe that RF has great potential in HUA prediction. A further prospective cohort is necessary for HUA developing prediction. Moreover, variables in this study only included common anthropometric and blood biomarkers. Other variables such as lifestyle, education background and income may also be integrated as a comprehensive epidemiological prediction model. This study is only based on the preliminary study of machine learning. We believe that it will provide a direction for the further study of machine learning in clinical research.

## Supplementary Material

Supplementary Material

## Data Availability

The datasets analyzed during the current study are available from the corresponding author on reasonable request.

## Abbreviations

BMI: body mass index
HUA: hyperuricemia
LR: logistic regression
NPV: negative predictive value
PPV: positive predictive value
RF: random forest
ROC: receiver operating characteristic

## References

[1] Ghei M., Mihailescu M. and Levinson D. (2002) Pathogenesis of hyperuricemia: recent advances. Curr. Rheumatol. Rep. 4, 270–274 10.1007/s11926-002-0076-z 12010614

[2] Chen-Xu M., Yokose C., Rai S.K., Pillinger M.H. and Choi H.K. (2019) Contemporary prevalence of gout and hyperuricemia in the United States and decadal trends: The National Health and Nutrition Examination Survey, 2007-2016. Arthritis Rheumatol. 71, 991–999 10.1002/art.40807 30618180 PMC6536335

[3] Liu R., Han C., Wu D., Xia X., Gu J., Guan H. et al. (2015) Prevalence of hyperuricemia and gout in Mainland China from 2000 to 2014: a systematic review and meta-analysis. Biomed. Res. Int. 2015, 762820 10.1155/2015/762820 26640795 PMC4657091

[4] Li Y., Shen Z., Zhu B., Zhang H., Zhang X. and Ding X. (2021) Demographic, regional and temporal trends of hyperuricemia epidemics in mainland China from 2000 to 2019: a systematic review and meta-analysis. Glob. Health Action. 14, 1874652 10.1080/16549716.2021.1874652 33475474 PMC7833047

[5] Bardin T. and Richette P. (2017) Impact of comorbidities on gout and hyperuricaemia: an update on prevalence and treatment options. BMC Med. 15, 123 10.1186/s12916-017-0890-9 28669352 PMC5494879

[6] Wu A.H., Gladden J.D., Ahmed M., Ahmed A. and Filippatos G. (2016) Relation of serum uric acid to cardiovascular disease. Int. J. Cardiol. 213, 4–7 10.1016/j.ijcard.2015.08.110 26341316

[7] Dalbeth N., House M.E., Aati O., Tan P., Franklin C., Horne A. et al. (2015) Urate crystal deposition in asymptomatic hyperuricaemia and symptomatic gout: a dual energy CT study. Ann. Rheum. Dis. 74, 908–911 10.1136/annrheumdis-2014-206397 25637002

[8] Puig J.G., Beltran L.M., Mejia-Chew C., Tevar D. and Torres R.J. (2016) Ultrasonography in the diagnosis of asymptomatic hyperuricemia and gout. Nucleosides Nucleotides Nucleic Acids 35, 517–523 10.1080/15257770.2015.1124999 27906639

[9] Cao J., Wang C., Zhang G., Ji X., Liu Y., Sun X. et al. (2017) Incidence and Simple Prediction Model of Hyperuricemia for Urban Han Chinese Adults: A Prospective Cohort Study. Int. J. Environ. Res. Public Health 14, 67 10.3390/ijerph14010067 PMC5295318 28085072

[10] Zeng J., Zhang J., Li Z., Li T. and Li G. (2020) Prediction model of artificial neural network for the risk of hyperuricemia incorporating dietary risk factors in a Chinese adult study. Food Nutr. Res. 64, 3712 10.29219/fnr.v64.3712 32047420 PMC6983978

[11] Lee S., Choe E.K. and Park B. (2019) Exploration of machine learning for hyperuricemia prediction models based on basic health checkup tests. J. Clin. Med. 8, 10.3390/jcm8020172 PMC6406925 30717373

[12] Zhang Q., Lou S.S., Meng Z.W. and Ren X.J. (2011) Gender and age impacts on the correlations between hyperuricemia and metabolic syndrome in Chinese. Clin. Rheumatol. 30, 777–787 10.1007/s10067-010-1660-7 21181218

[13] American Diabetes A (2021) 2. Classification and Diagnosis of Diabetes: Standards of Medical Care in Diabetes-2021. Diabetes Care 44, S15–S33 10.2337/dc21-S002 33298413

[14] Liu J., Tang Z.H., Zeng F., Li Z. and Zhou L. (2013) Artificial neural network models for prediction of cardiovascular autonomic dysfunction in general Chinese population. BMC Med. Inform. Decis. Mak. 13, 80 10.1186/1472-6947-13-80 23902963 PMC3735390

[15] Nathalie J. and Shaju S. (2002) The class imbalance problem: a systematic study. Intelligent Data Analysis 6, 429–449 10.3233/IDA-2002-6504

[16] Haibo H. and Garcia E.A. (2009) Learning from imbalanced data. IEEE Trans. Knowl. Data Eng. 21, 1263–1284 10.1109/TKDE.2008.239

[17] Rok B. and Lara L. (2010) Class prediction for high-dimensional class-imbalanced data. BMC Bioinformatics 11, 523 10.1186/1471-2105-11-523 20961420 PMC3098087

[18] Breiman L. (2001) Random Forests. Machine Learning 45, 5–32 10.1023/A:1010933404324

[19] Sugimoto K., Murata H., Hirasawa H., Aihara M., Mayama C. and Asaoka R. (2013) Cross-sectional study: Does combining optical coherence tomography measurements using the ‘Random Forest’ decision tree classifier improve the prediction of the presence of perimetric deterioration in glaucoma suspects? BMJ Open 3, e003114 10.1136/bmjopen-2013-003114 24103806 PMC3796272

[20] Kuo K.T., Chang Y.F., Wu I.H., Lu F.H., Yang Y.C., Wu J.S. et al. (2019) Differences in the association between glycemia and uric acid levels in diabetic and non-diabetic populations. J. Diabetes Complicat. 33, 511–515 10.1016/j.jdiacomp.2019.05.004 31176544

[21] Andrade J.A.M., Kang H.C., Greffin S., Garcia Rosa M.L. and Lugon J.R. (2014) Serum uric acid and disorders of glucose metabolism: the role of glycosuria. Braz. J. Med. Biol. Res. 47, 917–923 10.1590/1414-431X20143878 25250631 PMC4181228

[22] Ghasemi A. (2021) Uric acid-induced pancreatic beta-cell dysfunction. BMC Endocr Disord 21, 24 10.1186/s12902-021-00698-6 33593356 PMC7888074

[23] Mortada I. (2017) Hyperuricemia, Type 2 diabetes mellitus, and hypertension: an emerging association. Curr. Hypertens. Rep. 19, 69 10.1007/s11906-017-0770-x 28770533

