# Supplementary material for: Prediction model of random forest for the risk of hyperuricemia in a Chinese basic health checkup test
Source: Biosci Rep. 2021 Apr 7;41(4):BSR20203859. doi: 10.1042/BSR20203859 (PMC8026814; doi:10.1042/BSR20203859)
Supplement: Supplementary Material [file BSR-2020-3859_supp.pdf]

Supplementary Appendix

This supplementary material has been provided by the authors to give readers additional information about their work.

Appendix1 Twenty-one variables including anthropometric measurements, blood tests, alcohol intake, and smoking history were examined in the RF model. The table below shows all the included variables and their weight values in the model.

Supplementary Table 1. variables included in RF

male		female	
	**weight**		**weight**
TG	0.16137	TG	0.16429
Cr	0.14069	Cr	0.13386
ALT	0.09339	BMI	0.11987
BMI	0.08599	WC	0.07900
Weight	0.08226	ALT	0.07398
Age	0.06822	Weight	0.05659
WC	0.05596	BU	0.05639
TC	0.04578	Age	0.05294
FPG	0.04076	SBP	0.04263
WBC	0.03438	TC	0.03861
Height	0.03247	WBC	0.03009
RBC	0.03034	RBC	0.02662
BU	0.02841	Height	0.02464
TBIL	0.02779	FPG	0.02331
Hb	0.02285	DBP	0.02328
SBP	0.01659	Hb	0.02112
DBP	0.01384	Tbil	0.02078
Smoking	0.00546	UWBC	0.00604
Drinking	0.00490	URBC	0.00471
URBC	0.00477	Drinking	0.00117
UWBC	0.00380	Smoking	0.00008

TG = triglyceride, Cr = creatinine, ALT = alanine aminotransferase, BMI = body mass index, WC = waist circumference, TC = total cholesterol,FPG = fasting plasma glucose, WBC = white blood cell,RBC = red blood cell, BU= blood urea, TBIL = total bilirubin, Hb = hemoglobin, SBP = systolic blood pressure, DBP = diastolic blood pressure, URBC = urine red blood cell, UWBC = urine white blood cell.
